# Influence of dynamic preoperative body mass index changes on patient-reported outcomes after surgery for degenerative lumbar spine disease

**DOI:** 10.1007/s10143-020-01454-5

**Published:** 2020-12-11

**Authors:** Alessandro Siccoli, Marc L. Schröder, Victor E. Staartjes

**Affiliations:** 1 Department of Neurosurgery, Bergman Clinics, Amsterdam, The Netherlands; 2 Faculty of Medicine, University of Zurich, Zurich, Switzerland; 3 Department of Neurosurgery, Clinical Neuroscience Center, University Hospital Zurich, University of Zurich, Zurich, Switzerland; 4 Bergman Clinics, Naarden, Rijksweg 69, 1411 GE Naarden, The Netherlands

**Keywords:** Obesity, Overweight, Weight loss, Weight gain, Outcome

## Abstract

Psychological factors demonstrably and often massively influence outcomes of degenerative spine surgery, and one could hypothesize that preoperative weight loss may correlate with motivation and lifestyle adjustment, thus leading to potentially enhanced outcomes. We aimed to evaluate the effect of preoperative weight loss or gain, respectively, on patient-reported outcomes after lumbar spine surgery. Weight loss was defined as a BMI decrease of ≤ − 0.5 kg/m^2^ over a period of at least 1 month, and weight gain as a BMI increase of ≥ 0.5 kg/m^2^ in the same time period, respectively. The primary endpoint was set as the achievement of the minimum clinically important difference (MCID) in the ODI at 1 or 2 years postoperatively. A total of 154 patients were included. Weight loss (odds ratio (OR): 1.18, 95% confidence interval (CI): 0.52 to 2.80) and weight gain (OR: 1.03, 95% CI: 0.43 to 2.55) showed no significant influence on MCID achievement for ODI compared to a stable BMI. The same results were observed when analysing long-term NRS-BP and NRS-LP. Regression analysis showed no correlation between BMI change and PROM change scores for any of the three PROMs. Adjustment for age and gender did not alter results. Our findings suggest that both preoperative weight loss and weight gain may have no measurable effect on long-term postoperative outcome compared to a stable BMI. Weight loss preoperatively—as a potential surrogate sign of patient motivation and lifestyle change—may thus not influence postoperative outcomes.

## Introduction

Obesity is well known to be an independent risk factor for several comorbidities such as cardiovascular diseases or hypertension [24, 34]. Additionally, current literature suggests that obesity actively contributes to low back pain [11, 21, 42, 54], due to the effect of excessive weight on the lumbar spine, and thus consequently leads to a higher probability for that particular patient subpopulation to undergo lumbar spinal surgery [32]. The National Surgery Quality Improvement Program (NSQIP) database already stated that 44% of patients in a lumbar spine surgery cohort between 2005 and 2010 were obese [8], with an increasing tendency nowadays [1].

The effect of obesity on surgical outcome and complication rate has been well-investigated, with many studies identifying higher intraoperative and postoperative complication rates for obese patients [7, 12, 18, 33, 35]. This effect is first of all given by the higher prevalence of comorbidities, as well as post-surgical restricted activity [5, 10]. However, obesity itself also has an important effect on surgical outcome, possibly due to the higher mechanical load on the spine and therefore the surgical site [26]. Thirdly, there might be relevant biopsychosocial factors [13], as obesity has been shown to be a risk factor for depression [17], which itself is known to have an important impact on surgical outcome [9, 16, 45, 52].

However, the effect of weight on the surgical outcome is only well understood as a static value, whereas the effect of weight change prior to surgery remains unclear. In lumbar spine surgery, the effect of preoperative weight loss remains uncharted. As a result, studies analysing preoperative weight loss prior to lumbar spine surgery and its clinical effects have been called for [2, 21].

Nowadays, it is the general advice for overweight patients to lose weight prior to surgery, as these might profit from reduced mechanical stress and therefore higher chances for positive outcome [6, 23]. There may also be a psychological effect: Patients willing and able to lose weight prior to surgery may be more motivated for surgery and for post-surgical activity and may thus be hypothesized to experience enhanced outcomes [6]. Similarly, those patients who are motivated to lose weight preoperatively could be suffering from higher amounts of pain, thus potentially increasing likelihood of achieving a clinically important improvement.[4, 50]

The aim of this study is to determine the effect of dynamic weight loss or weight gain on surgical outcome in lumbar spine surgery, specifically on patient-reported outcome measures (PROMs).

## Materials and methods

### Design

From a prospective institutional registry of a single Dutch short-stay spine centre, all patients undergoing lumbar spine surgery between December 2010 and November 2019 were identified. All surgical procedures were performed by a single senior neurosurgeon (M.L.S.) as described previously [43, 47, 48, 51, 53]. Due to local restrictive regulations by insurance companies, patients aged > 80 or with a body mass index (BMI) > 33 or American Society of Anaesthesiologists (ASA) score > 2 cannot be considered for elective short-stay spine surgery [49]. We included only patients with complete BMI data as defined below, as well as complete baseline and 12-month or 24-month (i.e. long-term) PROMs, and without prior spine surgery at the index level [46].

### Ethical considerations

This registry was approved by the local institutional review board (Medical Research Ethics Committees United, Registration Number: W16.065). The study was performed according to the 2013 Declaration of Helsinki and its later amendments, and all patients provided written informed consent.

### Data collection

All surgical data and patient characteristics were systematically collected in a prospective registry. For all patients included in the study, weight and height were measured at the first preoperative visit and just before surgery. Patients were not informed about this study either pre- or postoperatively. To exclude short-term weight fluctuations, we included only patient with a minimum gap of 1 month between the first and the directly preoperative weight measurement, and BMI change was defined as a minimal difference of ≥ 0.5 kg/m^2^. All included patients were divided into 3 groups: Patients with a BMI decrease of − ≥ 0.5 kg/m^2^, those with a stable BMI, and those with a BMI increase of + ≥ 0.5 kg/m^2^. For baseline PROM assessment, all patients included completed a standardized questionnaire including a validated Dutch version of the Oswestry Disability Index (ODI) as a measure of functional disability, as well as a numeric rating scale (NRS) for leg pain and back pain severity [39]. Follow-up questionnaires with the same PROMs were automatically dispatched via e-mail using a validated follow-up system at 12 months and 24 months after surgery [39, 40]. If both 12- and 24-month PROMs were available for the same patient, the longer-term outcome was used in the statistical analysis [46].

### Statistical analysis

Categorical data are given as numbers (percentages) and continuous data as mean ± standard deviation.

The minimal clinically important difference (MCID), therefore clinical success, was set as ≥ 30% improvement [31] at 12-month or 24-month follow-up for ODI, or for NRS leg pain and NRS back pain, as both endpoints were shown to accurately reflect the other [46]. The primary endpoint of this study was long-term MCID in the ODI. For determining intergroup differences in MCID achievement, we conducted univariate logistic regression, where the patients with a stable BMI are defined as reference group, and additionally a multivariate logistic regression analysis adjusted for age and gender [44]. We also carried out univariate linear regression to evaluate the linear relationship between BMI change score and PROM change score and used multivariate linear regression for age and gender adjustment. All analyses were carried out using R Version 3.6.1 (The R Foundation for Statistical Computing, Vienna, Austria) [36]. A 2-tailed *p* ≤ 0.05 was considered statistically significant.

## Results

### Overview

**Fig. 1:**
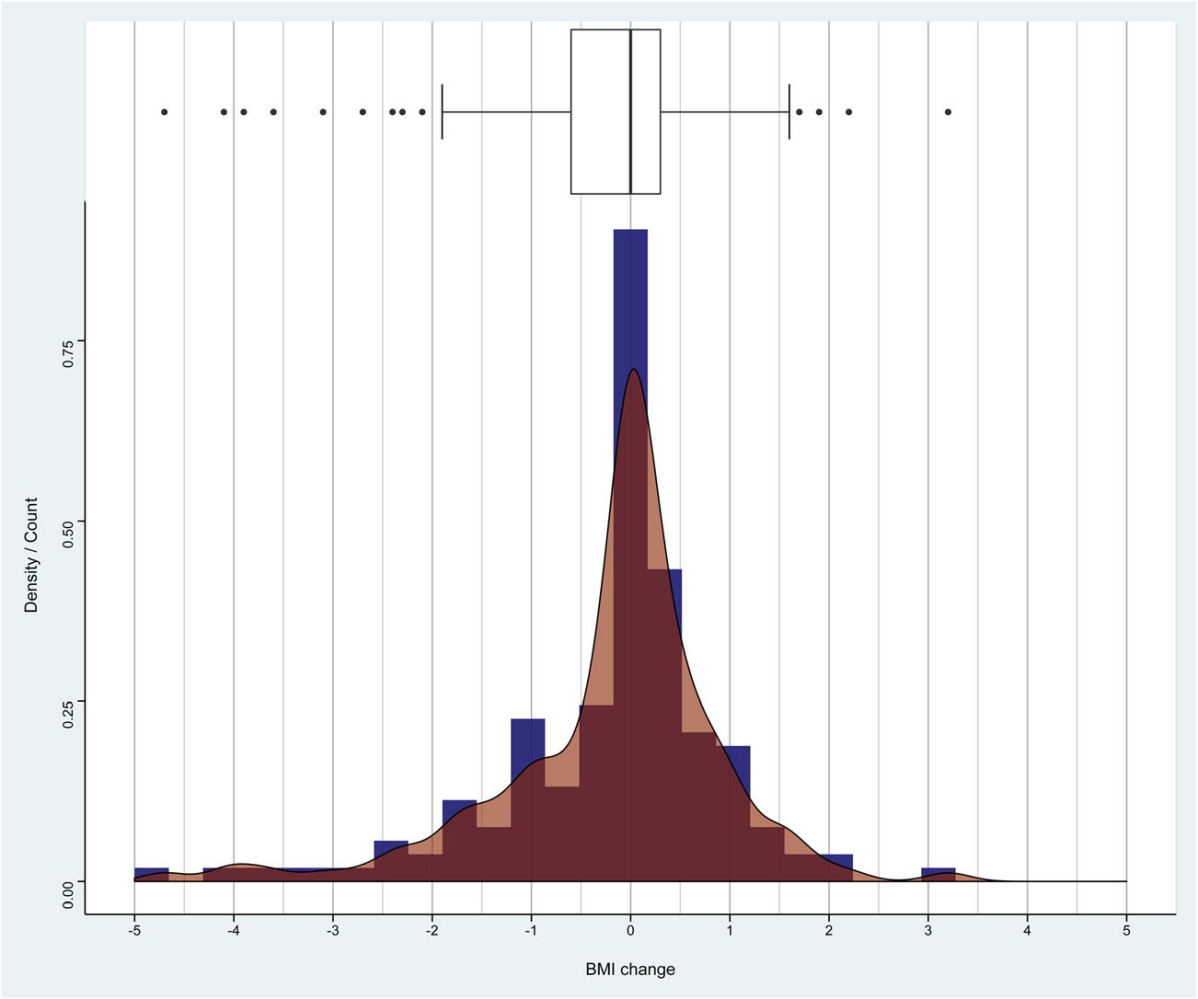
Distribution of BMI change score. The density plot (curve) demonstrates a non-parametric probability density function smoothed over the patient counts (bins), with the y-axis demonstrating the proportion of patients within these bins. The histogram demonstrates the distribution of patients among the timepoints. BMI, body mass index

**Fig. 2:**
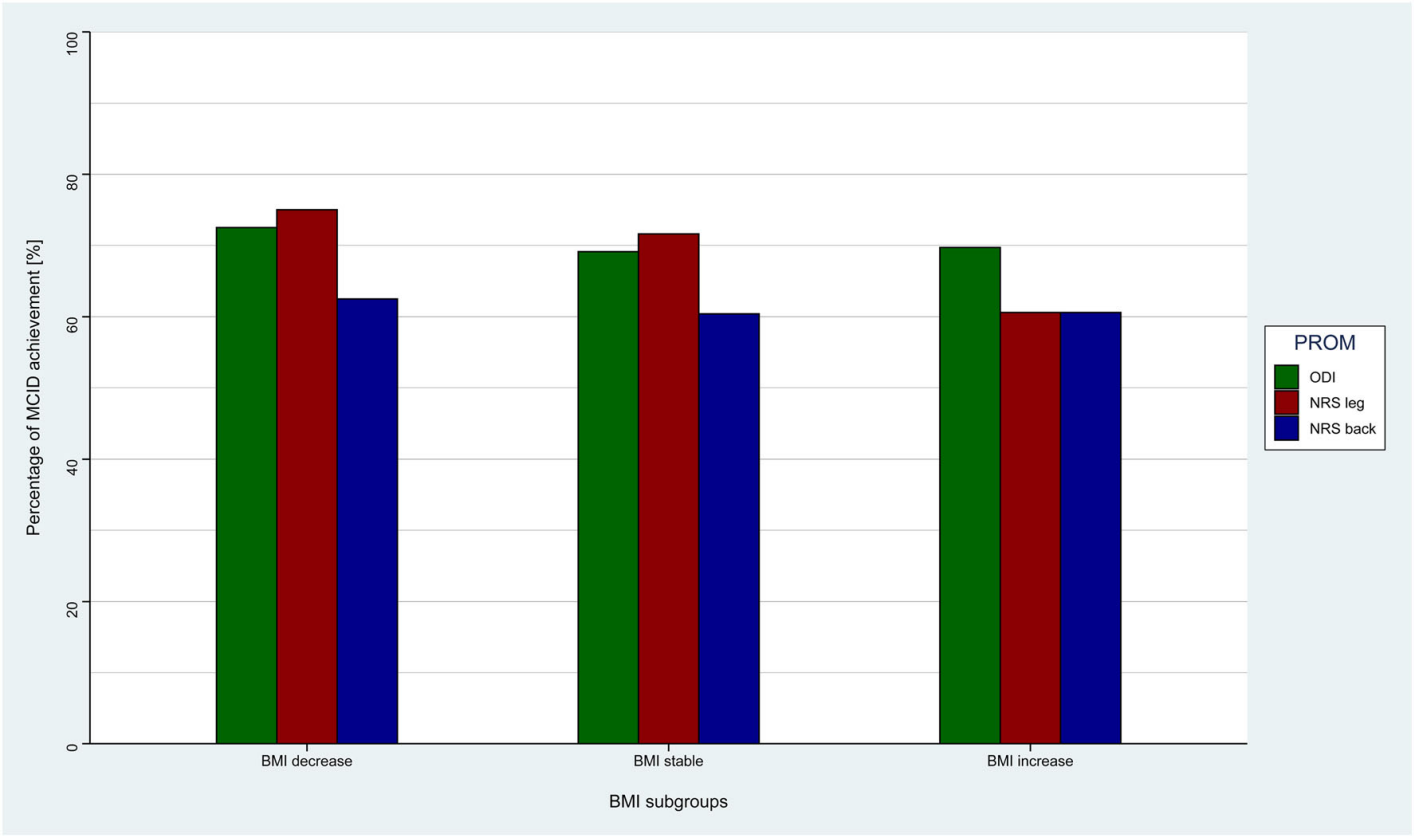
Distribution of MCID achievement percentages within all BMI subgroups for all 3 PROM values. BMI, body mass index; MCID, minimal clinically important difference; PROM, patient-reported outcome measurement; ODI, Oswestry Disability Index; NRS, numeric rating scale

Baseline characteristics along with surgical indications and baseline PROMs are reported in Table 1. In our prospective registry, 154 patients underwent lumbar spine surgery for degenerative disease and had complete PROM data and two different BMI values with at least a 1-month interval [40]. Most patients (87 pts., 56%) underwent tubular microdiscectomy, while 35 patients (23%) underwent decompression and interbody fusion and 32 (21%) underwent decompression only. We observed 40 (26%) patients with weight loss, 81 (53%) patients with stable BMI, and 33 (21%) patients with weight gain. The average BMI difference was − 0.18 ± 1.13 kg/m^2^, and mean inter-measurement interval was 121 ± 98 days. A bar and density plot with a corresponding box plot of the BMI change distribution is given in Fig. 1. Overall MCID achievement at long-term follow-up was 70.1% for ODI, 70.1% for NRS leg pain severity, and 61.0% for NRS back pain severity. The achievement rates for every BMI subgroup are shown in Fig. 2.

**Table 1:** Baseline patient characteristics

Characteristic	Value
Age	53.0 ± 11.9
Active smoker	46 (31%)
BMI (kg/m^2^)	26.2 ± 3.4
Indication	
LDH	87 (56%)
Lumbar stenosis	32 (21%)
DDD	19 (12%)
Spondylolisthesis	16 (10%)
Surgical technique	
Microdiscectomy	87 (56%)
Decompression and fusion	35 (23%)
Decompression	32 (21%)
ASA score	
Class I	72 (48%)
Class II	77 (52%)
Index level	
L1-L2	2 (1.3%)
L2-L3	4 (2.6%)
L3-L4	19 (12%)
L4-L5	83 (54%)
L5-S1	46 (30%)
Baseline ODI	44.5 ± 17.2
Baseline NRS leg	6.7 ± 2.4
Baseline NRS back	6.2 ± 2.5
Mean BMI change score	− 0.18 ± 1.13
Mean BMI measurement interval	121 ± 98

BMI, body mass index; LDH, lumbar disc herniation; DDD, degenerative disc disease; ASA, American Society of Anesthesiologists; ODI, Oswestry Disability Index, NRS, numeric rating scale

### Logistic regression

All results of the logistic regression are shown in Table 2. Compared to the patients with a stable BMI, weight loss (OR = 1.177, 95% CI = 0.516–2.797) or weight gain (OR = 1.027, 95% CI = 0.432–2.548) showed no statistical difference in MCID achievement for ODI—the primary endpoint. Regarding MCID for leg pain severity, the decrease in BMI (OR = 1.190, 95% CI = 0.511–2.912) or increase in BMI (OR = 0.610, 95% CI = 0.261–1.443) showed no difference compared to a stable BMI. The same result was obtained regarding MCID for back pain severity, where the decrease in BMI (OR = 1.088, 95% CI = 0.501–2.406) or increase in BMI (OR = 1.004, 95% CI = 0.441–2.336) did not influence long-term outcomes. After adjustment for age and gender, similar values were obtained without relevant changes in the direction or magnitude of the coefficients (all *p* > 0.05).

**Table 2:** Logistic regression analysis results of BMI increase or decrease compared to a stable BMI

Predictor	Odds ratio	CI (95%)	*p* value
ODI			
BMI decrease (“weight loss”)	1.18	0.52-2.80	0.704
BMI increase (“weight gain”)	1.03	0.43-2.55	0.953
NRS leg pain			
BMI decrease (“weight loss”)	1.19	0.51-2.91	0.693
BMI increase (“weight gain”)	0.61	0.26-1.44	0.254
NRS back pain			
BMI decrease (“weight loss”)	1.09	0.50-2.41	0.831
BMI increase (“weight gain”)	1.00	0.44-2.34	0.991

*p ≤ 0.05

CI, confidence interval; ODI, Oswestry Disability Index; NRS, numeric rating scale

### Linear regression

According to a linear regression analysis, there was no association among BMI change and ODI change scores (*p* = 0.923, *R*^2^ = − 0.007). The same result was obtained for NRS leg pain scores (*p* = 0.371, *R*^2^ = − 0.001), as well as for NRS back pain severity change scores (*p* = 0.385, *R*^2^ = − 0.002). After adjustment for gender and age, the results were preserved (ODI: *p* = 0.701 and *R*^2^ = − 0.010; NRS leg pain severity: *p* = 0.440 and *R*^2^ = − 0.002; NRS back pain: *p* = 0.438 and *R*^2^ = − 0.002).

## Discussion

We analysed 154 patients from a prospective registry who underwent lumbar spine surgery and who had dynamic BMI change data available throughout the preoperative period. A decrease in BMI of ≤ − 0.5 kg/m^2^ induced a minimal beneficial effect on functional and pain severity improvement, however not by statistical significance. An increase in BMI of ≥ 0.5 kg/m^2^ also showed no difference in MCID achievement for ODI, as well as for NRS leg pain and NRS back pain. Adjustment for age and gender did not influence results. Similarly, change scores of BMI and PROM change scores demonstrated no association. The hypothesis that dynamic weight loss in the preoperative period increases the benefit of surgery through biopsychosocial interactions is thus not supported by our data.

Overweight patients undergoing lumbar spine surgery are becoming more prevalent year by year [19]. Additionally, obesity has been shown to cause lumbar spinal degenerative disease, such as degenerative disc disease (DDD) with chronic low back pain or facet arthrosis [15, 22]. Therefore, considering body weight and obesity in treatment and prognosis of patients with spinal disorders is more and more a topic of major interest. Understanding the effect of obesity and especially of preoperative weight changes on surgical outcome is important and may pave the way for better decision-making—and thus higher rates of therapeutic success.

Studies investigating the effect of obesity on surgical outcome mainly show a worse outcome as well as a higher complication rate after surgery [29, 32]. Knutsson et al. [25] were able to show an association of BMI with not only worse surgical outcome but also with a lower level of patient satisfaction. In contradiction, Rihn et al. showed no difference between obese and non-obese patients in terms of postoperative outcome in lumbar discectomy [37]. Onyekwelu et al. also demonstrated that patient-reported outcomes were noninferior for the obese cohort in over 1000 patients undergoing surgery for lumbar spinal stenosis [30]. Thus, the literature on the effect of statically assessed obesity on surgical outcome is still contradictory. As a consequence, obesity should not currently be regarded as a strong negative risk factor for unfavourable outcome or even as a contraindication for degenerative lumbar spine surgery [3, 25, 29, 37].

However, clinical hypotheses may arise, linking a potential benefit in surgical outcome and satisfaction with dynamic weight loss—as opposed to static obesity—and concomitant lifestyle changes. It is well demonstrated that psychological factors such as depression and anxiety, as well as positive beliefs about surgery influence patient-reported outcomes after spine surgery [9, 27, 28, 52]. These factors may constitute a plausible mechanism that could link any potential effects of weight loss and lifestyle adjustments to patient-reported outcome. Additionally, for any degenerative musculoskeletal disorder, a reduction in mechanical load through weight loss could stimulate less chronic pain development, as has been demonstrated also in the spinal literature [14, 38].

Still, in contrast to static obesity before surgery or weight loss after surgery, there are only few studies investigating the dynamic effect of preoperative weight loss on postoperative surgical outcome, and none in the field of spinal science [6, 20, 23]. A study investigating the effect of preoperative weight loss on total knee arthroplasty outcome in obese patients with a BMI of ≥ 40 kg/m^2^ did not state any melioration in physical function improvement after weight loss [23].

Our study however did not show any statistically significant improvements in terms of PROMs related to weight loss. The local insurance restrictions not allowing surgery on obese people with a BMI > 33 kg/m^2^ may have weakened any potential beneficial effects of weight loss. Namely, these effects may be far more pronounced with greater or extreme weight loss or with weight loss in patients who are obese and then return to a normal or slightly overweight BMI preoperatively. Therefore, there might be a beneficial effect of preoperative weight loss on morbidly obese patients, while this effect might not be detectable in our patient cohort.

We found slightly but consistently higher odds ratios for MCID achievement in all of the three outcome measures for patients with preoperative weight loss, compared to those with a stable BMI. Nevertheless, the effect was too small to demonstrate statistical significance. Therefore, the presence of a benefit on surgical outcome after preoperative weight loss cannot be robustly ruled out by our findings and may only become statistically apparent in a larger patient cohort with greater statistical power. Even then, though, the effect would be weak. In the end, only a double-blinded randomized controlled trial could robustly identify such effects.

### Limitations

The main limitation of our study is its retrospective nature. Although all events were noted systematically and in a prospective patient registry, and all patients with sufficient data were included in this study, selection bias cannot be ruled out. Additionally, all data stem from a single centre and a single senior surgeon, from which the possibility of centre bias arises. Due to local insurance restrictions, only low-risk patients were allowed to be operated on—thus, those with a BMI < 33, ASA score 1 or 2, and age up to 80 years—meaning that our findings may not translate to morbidly obese or elderly patients or to patients with severe comorbidities. As discussed above, sample size considerations may constitute an additional limitation of our statistical analysis. We included patients who underwent more minor procedures such as decompression or microdiscectomy, as well as patients undergoing spinal fusion. Our sample size did not allow us to perform a powerful subgroup analysis according to these surgical factors. Also, following patients over a longer preoperative period—which would in turn allow larger weight changes—would certainly have been beneficial. Although most dietary and lifestyle weight loss studies show a significant weight loss after a month or less already, the highest weight loss is usually observed around 3 months into the intervention [41]. We had to opt for a 1-month minimum timespan because, at our centre, patients are usually operated within a few months after the first contact with the surgeon. Thus, we measured a mean time from the first to the last preoperative weight measurement of 121 ± 98 days (around 4 months). Lastly, we did not assess any psychological factors such as depression or anxiety, or postoperative weight changes, which could have elucidated the potential underlying mechanisms further.

## Conclusions

In an analysis of a large single-surgeon prospective registry, we found that both preoperative weight loss and weight gain had no measurable effect on long-term postoperative outcome compared to a stable BMI. However, we were unable to look at effects of preoperative weight loss in the morbidly obese (BMI > 33) population, as well as the effect of longer-term weight loss. Preoperative weight loss—as a potential surrogate sign of patient motivation and lifestyle change—may thus not influence postoperative outcomes. Further controlled and blinded studies are needed to fully comprehend the effect of weight and weight loss on postoperative outcomes, with higher statistical power and in a multicentre setting.

## Data Availability

The raw data will be provided upon reasonable request to the corresponding author.
